# Omics Reveals the Antibacterial Mechanism of Dihydromyricetin and Vine Tea Extract Against *Staphylococcus aureus* via Cell Wall and Membrane Disruption

**DOI:** 10.3390/molecules31020313

**Published:** 2026-01-16

**Authors:** Qiaoni Hui, Ting Li, Keke He, Wei Ma, Ying Guo, Yao Zhang, Liya Song

**Affiliations:** Department of Cosmetics, School of Light Industry Science and Engineering, Beijing Technology and Business University, Beijing 100048, China; huiqiaoni1111@163.com (Q.H.); qianandeliting@126.com (T.L.); 13121516520@163.com (K.H.); mawei901@163.com (W.M.); gy11072000@163.com (Y.G.); jiajingwenzhangyao@126.com (Y.Z.)

**Keywords:** *Staphylococcus aureus*, dihydromyricetin, vine tea extract, antibacterial mechanism, proteomics, lipidomics

## Abstract

*Staphylococcus aureus* (*S. aureus*) is a common pathogen that threatens healthcare and food safety. Vine tea extract (VTE) and its major active component, dihydromyricetin (DMY), show antibacterial activity. However, their mechanisms of action are not fully understood. In this study, we combined proteomics and lipidomics, with RT–qPCR validation of selected differentially expressed genes, to investigate how DMY and VTE affect *S. aureus*. Proteomics identified 210 and 535 differentially expressed proteins (DEPs) in the DMY-treated and VTE-treated groups, respectively. These DEPs were mainly enriched in cell wall- and membrane-associated pathways. DMY markedly increased proteins involved in fatty acid degradation, glyceride metabolism, and cell wall synthesis. In contrast, VTE increased proteins related to heme/iron acquisition and cell wall degradation. In addition, VTE altered proteins involved in pyrimidine metabolism and aminoacyl-tRNA biosynthesis, suggesting that non-DMY components in VTE may contribute to the antibacterial activity through additional pathways. Lipidomics further indicated membrane lipid remodeling, including increased fatty acid unsaturation and shorter acyl chain length. Collectively, DMY and VTE may inhibit *S. aureus* growth by remodeling membrane lipids and disturbing cell wall–cell membrane homeostasis. These findings provide mechanistic support for further development of DMY and VTE as natural antimicrobial candidates.

## 1. Introduction

*S. aureus* is a common foodborne pathogen [1,2,3] and an opportunistic pathogen [4,5,6], causing infections that range from localized skin and soft tissue infections [7,8] to severe invasive systemic infections [9,10,11]. However, the ecological risks, potential health hazards, and increasing bacterial resistance associated with conventional antimicrobial agents have raised significant concerns. Consequently, the development of novel natural antimicrobial agents has become an important research focus in both food preservation and medical applications [12].

Ampelopsis grossedentata, commonly known as vine tea, is a perennial woody vine mainly distributed in southern China. Because of its medicinal value, the leaves and young stems have been consumed as a traditional herbal tea for hundreds of years. In traditional medicine, vine tea is widely used to prevent and relieve symptoms such as colds, fever, sore throat, and toothache [13]. Modern studies have shown that vine tea has multiple biological activities, including antioxidant [14,15], antitumor [16,17], antidiabetic [18,19], antiviral [20], anti-inflammatory [21], and antibacterial effects [22]. Vine tea has a complex chemical composition, and about 40 unique compounds have been identified to date. Dihydromyricetin (DMY) is considered its major bioactive component. DMY is a dihydroflavonol with diverse pharmacological activities, including anti-inflammatory, antioxidant, antitumor, and immunomodulatory effects [23]. DMY shows antibacterial activity against several pathogens, including *S. aureus*, *Shigella flexneri*, and *Pseudomonas aeruginosa* [24,25].

The cell wall and membrane constitute the first line of defense against antimicrobial agents in bacteria. Our previous studies also showed that DMY and VTE can inhibit *S. aureus* by compromising cell wall integrity and increasing membrane permeability [26]. However, the underlying molecular mechanisms remain unclear. Based on these results, we combined proteomics [27], lipidomics [28], and RT-qPCR to identify key proteins and lipid alterations and to elucidate the key pathways associated with the antibacterial effects of DMY and VTE. This work may support the development of natural antimicrobial agents for food preservation and related applications.

## 2. Results and Discussion

### 2.1. Characterization of Major Flavonoids in Vine Tea Extract

**Figure 1:**
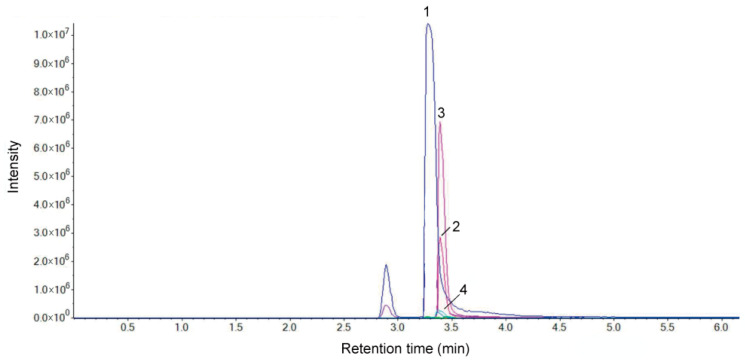
Overlaid MRM chromatograms of VTE: Peak 1: dihydromyricetin; Peak 2: dihydroquercetin; Peak 3: myricetin-3-O-rhamnoside; Peak 4: myricetin.

We characterized the major flavonoids in VTE using liquid chromatography-tandem mass spectrometry (LC-MS/MS). The overlaid multiple reaction monitoring (MRM) chromatograms are shown in Figure 1. Based on the LC-MS/MS data (Figure 1 and Table 1), VTE contained a relatively high level of flavonoids. Four major flavonoids were detected and identified: dihydromyricetin, dihydroquercetin, myricetin 3-O-rhamnoside, and myricetin. Among them, DMY was the predominant bioactive component.

### 2.2. Identification of DEPs

**Figure 2:**
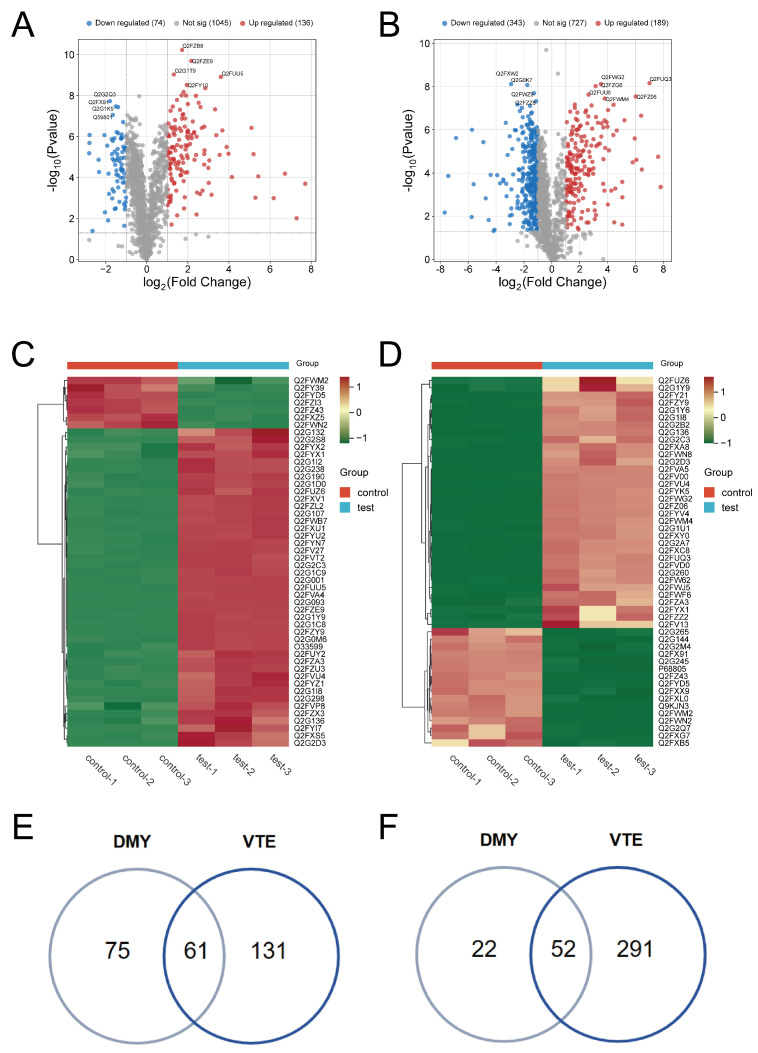
DEPs in *S. aureus* after DMY and VTE treatments. (**A**) Volcano plot of DEPs in DMY-treated *S. aureus*. (**B**) Volcano plot of DEPs in VTE-treated *S. aureus*. (**C**) Hierarchical clustering heatmap of DEPs in DMY-treated *S. aureus*. (**D**) Hierarchical clustering heatmap of DEPs in VTE-treated *S. aureus*. (**E**) DEPs jointly upregulated in DMY-treated and VTE-treated groups. (**F**) DEPs jointly downregulated in DMY-treated and VTE-treated groups.

A total of 1257 proteins were identified in the DMY group and 1259 proteins in the VTE group. Differentially expressed proteins (DEPs) were screened using a threshold of *p* ≤ 0.05 and fold change ≥ 2. Using these criteria, 210 DEPs were identified in the DMY-treated group compared with the control. Among them, 136 proteins were significantly upregulated and 74 were significantly downregulated (Figure 2A). In the VTE-treated group, 535 DEPs were identified relative to the control group, including 192 upregulated and 343 downregulated proteins (Figure 2B). Figure 2C,D show the overall expression patterns of DEPs in *S. aureus* after DMY and VTE treatment. In addition, 61 DEPs were co-upregulated (Figure 2E) and 52 DEPs were co-downregulated (Figure 2F).

### 2.3. Functional Annotation and Pathway Enrichment

**Figure 3:**
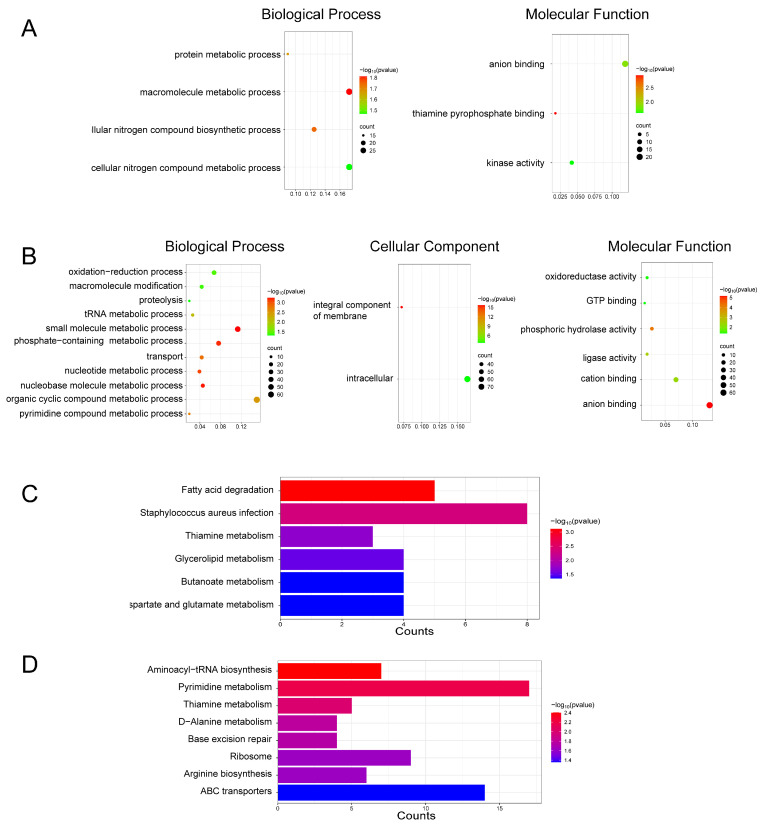
Functional enrichment of DEPs induced by DMY and VTE treatments in *S. aureus*. (**A**) GO enrichment results for the DMY group. (**B**) GO enrichment results for the VTE group. (**C**) KEGG pathway enrichment results for the DMY group. (**D**) KEGG pathway enrichment results for the VTE group.

GO enrichment analysis showed that the DEPs in the DMY group were mainly involved in several biological processes (BP), including protein metabolism, macromolecule metabolism, and the biosynthesis of cellular nitrogen compounds. In terms of molecular function (MF), these DEPs were primarily associated with anion binding and kinase activity, among other functions (Figure 3A). KEGG pathway enrichment analysis further revealed that DEPs in the DMY group were significantly enriched in pathways related to fatty acid degradation, glyceride metabolism, amino acid metabolism, and butyrate metabolism (Figure 3C). Taken together, the GO and KEGG results suggest that the antibacterial activity of DMY may be associated with the regulation of fatty acid degradation and glycerol metabolism pathways. Such metabolic alterations may disrupt cell membrane structural stability.

GO enrichment analysis of DEPs in the VTE group indicated enrichment in several key biological processes (BP), including ribosome biogenesis, pyrimidine metabolism, non-coding RNA metabolism, tRNA metabolism, and nucleotide metabolism. These DEPs were also associated with multiple cellular components (CC), such as the integral component of membranes and intracellular components. The molecular functions (MF) of DEPs in the VTE group included phospholipase activity, ligase activity, cation binding, and anion binding (Figure 3B). KEGG pathway analysis further showed that these DEPs were significantly enriched in aminoacyl-tRNA biosynthesis, pyrimidine metabolism, and D-alanine metabolism (Figure 3D). Taken together, the GO and KEGG results suggest that VTE affected cell wall- and membrane-associated processes in a manner similar to DMY. In addition, components other than DMY in VTE may contribute to growth inhibition of *S. aureus* through additional pathways. For example, disruption of pyrimidine metabolism may affect DNA/RNA biosynthesis, and interference with aminoacyl-tRNA biosynthesis may impair protein synthesis. Collectively, these pathways may underlie the multi-target antibacterial activity of VTE.

In summary, GO and KEGG enrichment analyses suggest that DMY and VTE exert their antibacterial effects mainly by disrupting cell wall and membrane structures. In addition, DMY may affect amino acid metabolism, whereas non-DMY components in VTE may influence pyrimidine metabolism and aminoacyl-tRNA biosynthesis. Together, these changes may contribute to the antibacterial activity.

### 2.4. DEPs Associated with the Cell Wall and Cell Membrane in the DMY-Treated Group

#### 2.4.1. DEPs Associated with the Cell Wall

The bacterial cell wall is essential for protection, maintenance of cell shape, and exchange of materials with the environment. Disruption of the cell wall can cause cell death. Many antibiotics, including penicillin, act by targeting cell wall synthesis or integrity [29]. In Gram-positive bacteria, the cell wall is mainly composed of peptidoglycan and teichoic acids. After DMY treatment of *S. aureus*, several proteins related to cell wall metabolism were significantly upregulated. These included LytM, SceD, and LtaS, which increased by 7.22-fold, 2.68-fold, and 9-fold, respectively. LytM is a Zn^2+^-dependent glycine–glycine endopeptidase secreted by *S. aureus*. It belongs to the staphylococcal lysostaphin-type metallopeptidase family [30]. LytM functions as a lytic enzyme and contributes to cell wall remodeling and autolysis by hydrolyzing peptidoglycan. It cleaves glycine–glycine peptide bonds in *S. aureus* peptidoglycan, which can weaken cell wall integrity [31,32]. SceD is a glycosyltransferase that also shows cell wall hydrolase activity [33]. It cleaves the β-1,4-glycosidic bond between N-acetylmuramic acid and N-acetylglucosamine in peptidoglycan (PG) [34]. This activity affects PG turnover and septum separation during cell division [35]. LtaS is a key enzyme in lipoteichoic acid (LTA) biosynthesis [36,37]. LTA is a major component of Gram-positive cell walls, and loss of LTA is typically associated with defects in cell division and reduced growth. DMY exposure may induce cell wall stress or damage. Under these conditions, increased expression of LytM and SceD could promote further hydrolysis of damaged PG peptide and glycosidic bonds. The upregulation of LtaS may indicate an adaptive response aimed at maintaining or restoring cell wall structure.

#### 2.4.2. DEPs Associated with the Cell Membrane

The bacterial cell membrane mainly consists of glycerophospholipids and proteins. After DMY treatment, 16 membrane-associated proteins increased in abundance, whereas 6 decreased. These proteins were mainly related to glyceride metabolism, fatty acid degradation, and other membrane-associated functions.

**Figure 4:**
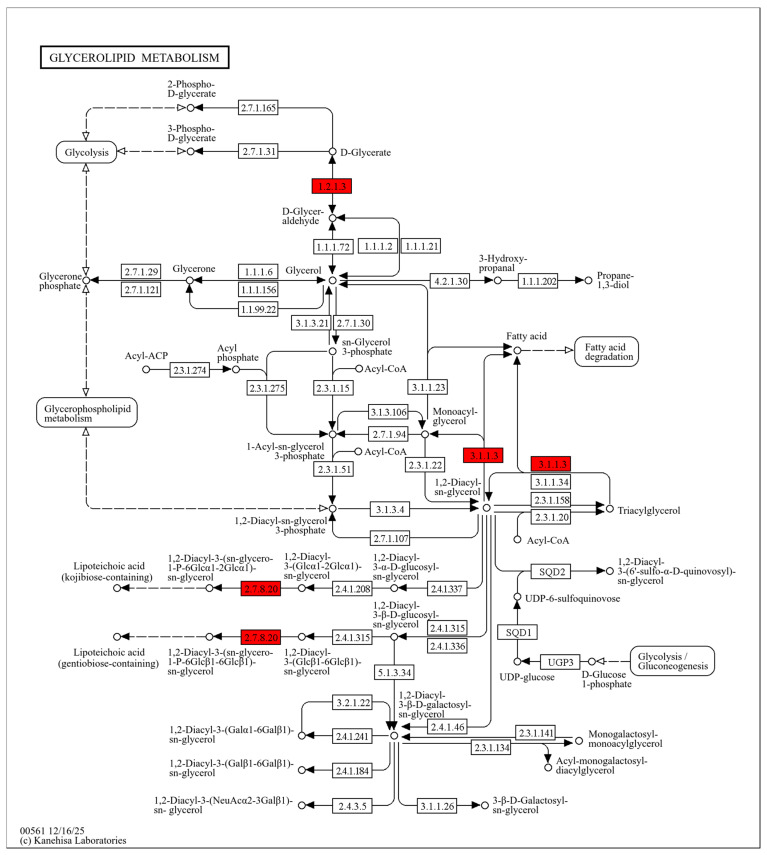
Glycerolipid metabolism pathway map in *S. aureus* after DMY treatment. Upregulated DEPs are highlighted in red.

Glycerides are essential cellular lipids with important physiological roles. They act as major energy storage molecules (e.g., triacylglycerols) and provide the structural basis for membrane lipids (e.g., phospholipids) [38]. In this study, three glyceride metabolism–related proteins were significantly upregulated after DMY treatment, including ALDH (EC 1.2.1.3), Lipase 1, and Lipase 2 (EC 3.1.1.3). (Figure 4) ALDH can catalyze the conversion of D-glyceraldehyde to D-glyceric acid [39]. Previous studies have shown that oxidative stress and membrane lipid peroxidation triggered by antibacterial agents can generate reactive aldehydes that are toxic to cells. Bacteria can reduce this toxicity by converting aldehydes into less harmful forms via ALDH-mediated “acidification” [40]. Therefore, the upregulation of ALDH suggests that DMY may induce lipid peroxidation in *S. aureus*. In this context, glycerol oxidation may produce glyceraldehyde, and increased ALDH activity may help mitigate aldehyde-associated toxicity. Glyceraldehyde can also be further converted to D-glyceric acid, which may reduce its intracellular accumulation. Lipases hydrolyze triacylglycerols into diacylglycerols (DAG), which can be further converted into monoacylglycerols (MAG), ultimately yielding glycerol and free fatty acids. In *S. aureus*, Lipase 1 and Lipase 2 belong to the same lipase family and show similar functional properties. Glycerol and fatty acids are key precursors for phospholipid synthesis, and phospholipids are major structural components of bacterial membranes [41,42]. Under DMY exposure, membrane lipids may be disrupted, and damaged membrane lipids may be further degraded by lipases to release fatty acids.

**Figure 5:**
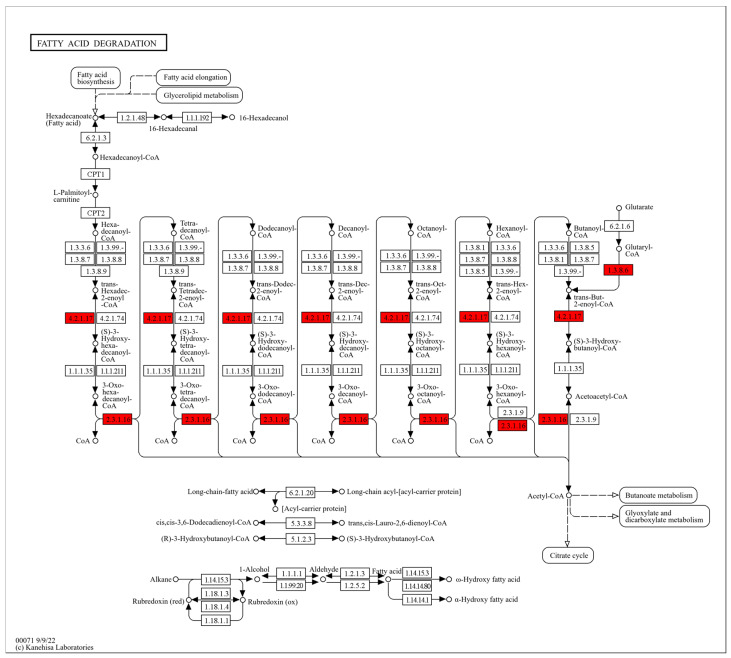
Fatty acid degradation pathway map in *S. aureus* after DMY treatment. Upregulated DEPs are highlighted in red.

Fatty acids are not only major energy substrates but also essential building blocks of phospholipids. Therefore, changes in fatty acid metabolism are closely associated with the physical properties of cell membranes, including rigidity and fluidity [43]. In our study, DMY treatment markedly upregulated several key enzymes in the fatty acid degradation pathway of *S. aureus*, including GCDH (EC 1.3.8.6), ECH (EC 4.2.1.17), and ACAA1 (EC 2.3.1.16), with fold changes of 1.45, 2.67, and 6.97, respectively (Figure 5). GCDH catalyzes the oxidation and decarboxylation of glutaryl-CoA, which is generated during fatty acid degradation, producing trans-2-enoyl-CoA and CO_2_ [44]. ECH then hydrates trans-2-enoyl-CoA to form 3-hydroxyacyl-CoA [45,46]. This intermediate is subsequently oxidized to 3-ketoacyl-CoA and cleaved by ACAA1 to generate acetyl-CoA and acyl-CoA. Together, these enzymes facilitate the conversion of fatty acid-derived intermediates into acetyl-CoA. These results suggest that DMY exposure may damage the bacterial cell membrane, promoting lipase-mediated hydrolysis of membrane lipids into fatty acids. The released fatty acids are then further degraded into acetyl-CoA through the enzymes described above. As a central metabolic intermediate, acetyl-CoA supports cellular energy metabolism and may provide energy for repairing damaged cell wall and membrane structures. In addition, acetyl-CoA is a key precursor for fatty acid biosynthesis and can contribute to the synthesis of new fatty acids to replenish impaired membranes [47].

#### 2.4.3. DEPs Associated with Membrane Proteins

Cell membrane proteins are essential for bacterial survival under drug pressure. They enable nutrient acquisition, maintain ion and energy homeostasis, export toxic compounds, and support stress responses [48]. After DMY treatment, both ABC transporters and phosphotransferase (PTS) systems in the cell membrane of *S. aureus* were significantly upregulated. ABC transporters mediate the influx and efflux of a wide range of substrates, including amino acids, inorganic ions, carbohydrates, complex organic molecules, and toxic compounds. Notably, certain ABC efflux pumps can export antibiotics [49,50]. During bacterial growth and division, peptidoglycan (PG) undergoes continuous remodeling, degradation, and recycling [51]. Accordingly, ABC transporters can influence PG biosynthesis and remodeling. For example, in Bacillus subtilis, overexpression of the YtrBCDEF ABC transporter markedly alters PG biosynthesis and results in a thicker PG layer [52]. In addition, the BceAB-type ABC transporter has been proposed to protect PG synthesis through a “target protection/rescue” mechanism, releasing undecaprenyl pyrophosphate (UPP) from bacitracin inhibition [53]. PTS are multicomponent sugar transporters responsible for importing various sugars and improving carbon source uptake [54]. Therefore, the DMY-induced upregulation of ABC transporters and PTS is expected to enhance sugar uptake and the efflux of harmful substances, and may also promote cell wall synthesis.

### 2.5. DEPs Associated with the Cell Wall and Cell Membrane in the VTE-Treated Group

Because DMY is a major active component of VTE, comparing the shared and distinct DEPs induced by these two agents is informative. This comparison helps clarify the common antibacterial mechanisms of VTE and identify pathways that may be specific to VTE. In this section, we first describe cell wall/cell membrane–associated proteins that were upregulated by both treatments. We then examine VTE-specific changes to infer potential contributions from non-DMY components. To emphasize proteins with larger expression differences, we used stricter criteria (unique peptides > 2, *p* ≤ 0.05, and fold change ≥ 3). Under these criteria, DMY treatment yielded 74 DEPs, including 18 proteins that function in or localize to the cell wall or cell membrane [36,55,56,57]. VTE treatment yielded 192 DEPs, including 34 proteins that directly function in or localize to the cell wall or cell membrane [33,58,59,60]. Cross-comparison identified seven shared proteins: LytM, LtaS, an ABC transporter, SceD, a PTS system component, ClfB, and Lipase 1. All seven proteins were significantly upregulated after both DMY and VTE treatments. Among them, LytM, LtaS, and Lipase 1 showed larger changes in the DMY-treated group.

We also identified VTE-specific cell wall/cell membrane–associated DEPs, with IsdE and ArlR showing the most pronounced alterations. IsdE is an ATP-dependent heme-binding ABC transporter that transfers heme across the cytoplasmic membrane. The imported heme can then be processed by IsdG/IsdI to release iron [61]. The increased abundance of IsdE after VTE exposure may indicate an adaptive response to impaired heme/iron acquisition under cell envelope stress. ArlR regulates the cell wall by affecting the expression of autolysis-related genes, and its upregulation may promote LytM expression [62]. Higher levels of autolysis-related factors may accelerate peptidoglycan turnover and weaken the cell wall.

### 2.6. DEPs Associated with Pyrimidine Metabolism in the VTE-Treated Group

In the VTE-treated group, we observed marked changes not only in cell wall/cell membrane–related proteins but also in proteins involved in pyrimidine metabolism. Five proteins were downregulated (DHODH, Ndk, CTPS, Cdd, and UPP), while one protein was upregulated (TSase). Pyrimidine nucleotides are required for DNA and RNA synthesis. Pyrimidine derivatives and their analogs can inhibit DNA replication and interfere with other essential cellular functions, thereby producing bacteriostatic effects [63].

In this pathway, DHODH catalyzes the conversion of dihydroorotate to orotate [64]. Orotate is then converted to UMP by enzymes such as UMPS. UMP is further phosphorylated to UDP and UTP by UMP-CMP kinase and Ndk [65]. UTP is a substrate for CTPS, which uses glutamine to generate CTP [66,67]. CTP is required for RNA synthesis and also serves as a precursor for membrane phospholipid biosynthesis [68]. CTP can be degraded to CDP, CMP, or cytidine (Cyd). Cdd catalyzes cytidine deamination to form uridine (Urd), which supports pyrimidine salvage and recycling [69]. UPP synthesizes UMP from uridine and PRPP, providing precursors for RNA synthesis [70]. Ndk and Cdd also participate in deoxynucleotide metabolism. After VTE exposure, the strong downregulation of DHODH, Ndk, CTPS, Cdd, and UPP suggests that pyrimidine nucleotide synthesis, activation, and recycling were impaired. This would limit the supply of nucleotides needed for bacterial DNA and RNA synthesis. TSase is the only enzyme that catalyzes the reduction of dUMP to dTMP. dTMP can be further phosphorylated to dTTP, which is essential for DNA synthesis [71]. The significant upregulation of TSase after VTE treatment may indicate a compensatory response to disturbed DNA synthesis. Under this condition, bacteria may increase de novo thymidylate synthesis by elevating TSase to support DNA repair.

Overall, non-DMY components in VTE (including apigenin, dihydroquercetin, quercetin, and gardenoside) may contribute to growth inhibition by downregulating DHODH, Ndk, CTPS, Cdd, and UPP. These changes could suppress de novo synthesis, energy-dependent activation, and salvage/recycling of pyrimidine nucleotides, thereby restricting DNA and RNA synthesis. Although TSase was upregulated and may help maintain thymidine supply during DNA repair, the broader metabolic disruption is still likely to hinder bacterial growth [72,73].

### 2.7. DEPs Related to Aminoacyl-tRNA Biosynthesis in the VTE-Treated Group

VTE may also affect DEPs involved in aminoacyl-tRNA biosynthesis. After VTE treatment, we identified six downregulated proteins associated with amide-tRNA formation: GlyRS, ArgRS, CysRS, HisRS, LysRS, and the C subunit of aspartyl/glutamyl-tRNA (Asp/Glu) amidotransferase. Aminoacyl-tRNA formation is the covalent attachment of a specific amino acid to the 3′ end of its corresponding tRNA, catalyzed by aminoacyl-tRNA synthetases (aaRS). Aminoacyl-tRNAs are the direct substrates for ribosome-mediated translation [74] and are essential for protein biosynthesis.

GlyRS, ArgRS, CysRS, HisRS, and LysRS catalyze the charging of glycine, arginine, cysteine, histidine, and lysine onto their respective tRNAs [75,76]. Therefore, the downregulation of these five enzymes after VTE exposure may decrease the corresponding aminoacyl-tRNA pools and suppress protein synthesis in Staphylococcus aureus [77]. The C subunit of aspartyl/glutamyl-tRNA (Asn/Gln) amidotransferase is required for amide-tRNA maturation and helps correct mis-amidated tRNAs, thereby ensuring accurate aminoacyl-tRNA formation [78]. This subunit was significantly downregulated after VTE exposure. This change may weaken its proofreading capacity and increase the accumulation of mis-acylated tRNAs. As a result, acyl-tRNA maturation may be impaired, further disrupting protein synthesis in *S. aureus* [79].

In summary, in addition to its effects on the cell wall and cell membrane of *S. aureus*, VTE also perturbs pyrimidine metabolism and aminoacyl-tRNA synthesis. These broader changes suggest a more complex antibacterial mechanism than DMY alone [80].

### 2.8. Lipidomics Analysis

Proteomic analysis indicated that DMY and VTE affect the cell wall and membrane of *S. aureus*. To further evaluate these effects, we investigated how DMY and VTE alter the lipid composition of *S. aureus*. Compared with the control group, *S. aureus* cells treated with DMY and VTE showed significant changes in lipid composition.

#### 2.8.1. Effects of DMY and VTE on Lipid Composition in *S. aureus*

**Figure 6:**
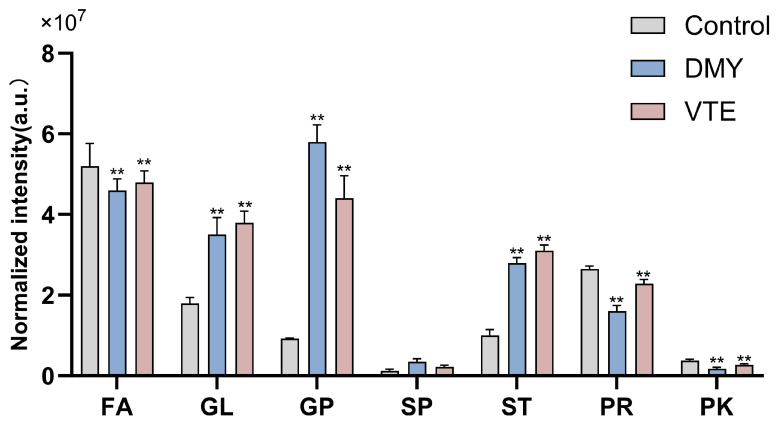
Relative lipid abundance in *S. aureus* after treatment with DMY and VTE. **: *p* < 0.01.

Our lipidomics analysis showed that the lipid classes with higher relative abundance in *S. aureus* were, in descending order, fatty acids (FA), prenyl lipids (PR), glycerides (GL), sterol lipids (ST), glycerophospholipids (GP), polyketides (PK), and sphingolipids (SP). Compared with the control group, the DMY- and VTE-treated groups showed significantly higher relative levels of glycerides, glycerophospholipids, sterol lipids, and sphingolipids (*p* < 0.01). In contrast, the relative levels of fatty acids, prenyl lipids, and polyketides were significantly lower (*p* < 0.01) (Figure 6). This observation is consistent with the marked upregulation of GCDH, ECH, and ACAA1, which are involved in fatty acid degradation.

#### 2.8.2. Effects of DMY and VTE on Glycerophospholipids

##### Saturated and Unsaturated Fatty Acids in Phospholipids

Our results showed that DMY and VTE treatment significantly increased the degree of unsaturation of phospholipid fatty acids in *S. aureus*. Previous studies on membrane fluidity in Gram-positive bacteria have mainly focused on regulation of fatty acid composition, including the production of unsaturated fatty acids, changes in fatty acid chain length, and shifts in the proportion of branched-chain fatty acids [81].

**Figure 7:**
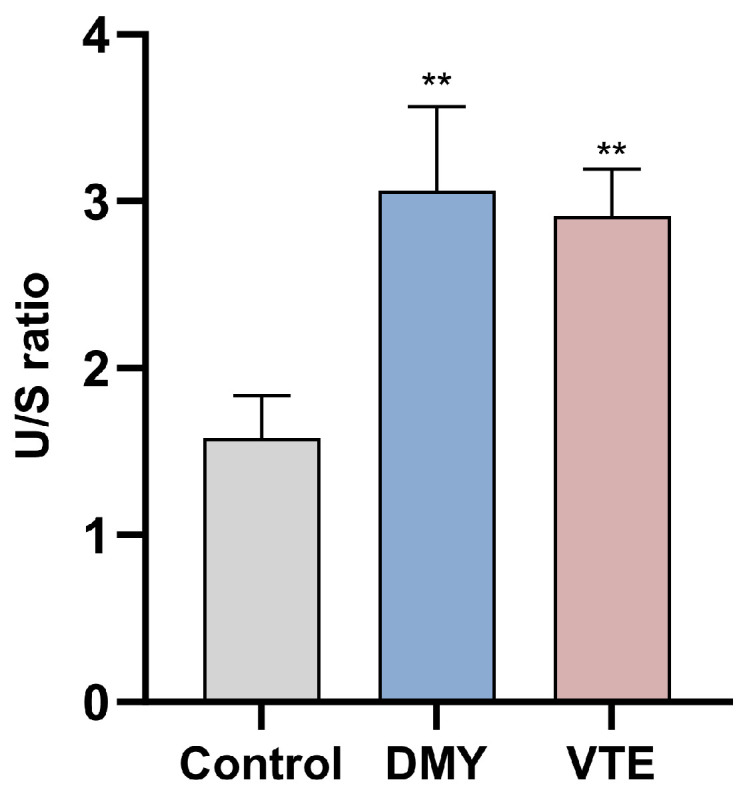
Unsaturated/saturated (U/S) ratio of phospholipid fatty acids in *S. aureus* after DMY and VTE treatment. **: *p* < 0.01.

Unsaturation is defined as the ratio of unsaturated to saturated fatty acids in cell wall- and membrane-associated phospholipids, and it is an important indicator of membrane fluidity. Higher unsaturation generally indicates greater membrane fluidity [82]. In this study, DMY and VTE increased phospholipid fatty acid unsaturation from 1.58 in the control group to 3.06 and 2.91, respectively (*p* < 0.01) (Figure 7), indicating that both treatments may increase membrane fluidity.

##### Carbon Chain Length of Phospholipid Fatty Acids

**Figure 8:**
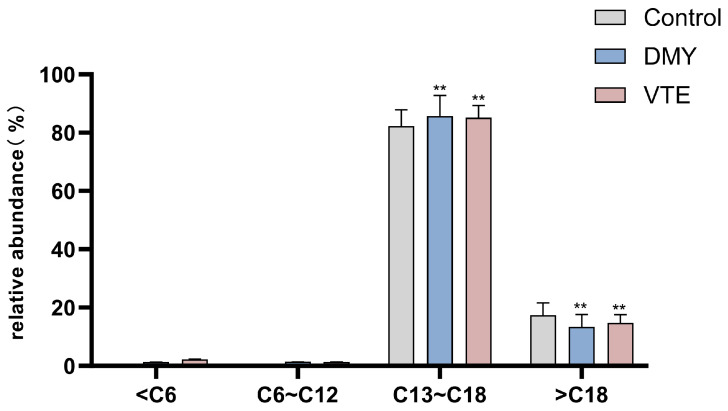
Relative abundance of fatty acids with different carbon chain lengths in *S. aureus* after DMY and VTE treatment. **: *p* < 0.01.

The chain length of phospholipid fatty acids is an important determinant of membrane fluidity [83]. After DMY and VTE treatment, the proportion of very long-chain fatty acids in *S. aureus* decreased from 17.36% in the control group to 13.36% and 14.70%, respectively (*p* < 0.01) (Figure 8). In contrast, the proportion of long-chain fatty acids increased from 82.21% to 85.70% and 85.09%, respectively (*p* < 0.01). These results suggest that DMY and VTE shorten phospholipid fatty acid chain length in the cell wall and membrane of *S. aureus*, which may increase membrane fluidity.

#### 2.8.3. Effects of DMY and VTE on Glycerolipids

**Figure 9:**
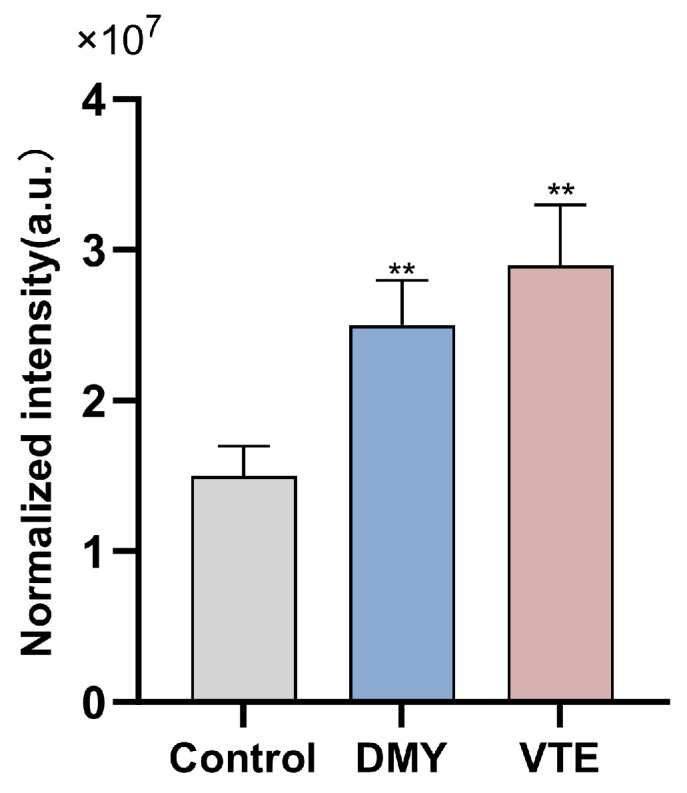
Relative abundance of DAG in *S. aureus* after DMY and VTE treatment. **: *p* < 0.01.

Previous studies have reported that diacylglycerol (DAG) is a major glycerolipid metabolite in bacteria. Several studies also indicate that abnormal intracellular accumulation of DAG can significantly inhibit bacterial growth, although the underlying mechanism remains unclear [84]. In our study, the relative DAG abundance in the control, DMY-treated, and VTE-treated groups was 1.5 × 10^7^, 2.5 × 10^7^, and 2.9 × 10^7^, respectively (Figure 9). DAG levels were significantly higher in both treatment groups than in the control group (*p* < 0.01), which may be associated with growth inhibition. The DAG increase may be related to the upregulation of LtaS observed above. LtaS promotes the formation of DAG-related intermediates during lipoteichoic acid synthesis, and DAG accumulation may contribute to antibacterial effects [36,37].

#### 2.8.4. Effects of DMY and VTE on Isoprenoid Lipids

**Figure 10:**
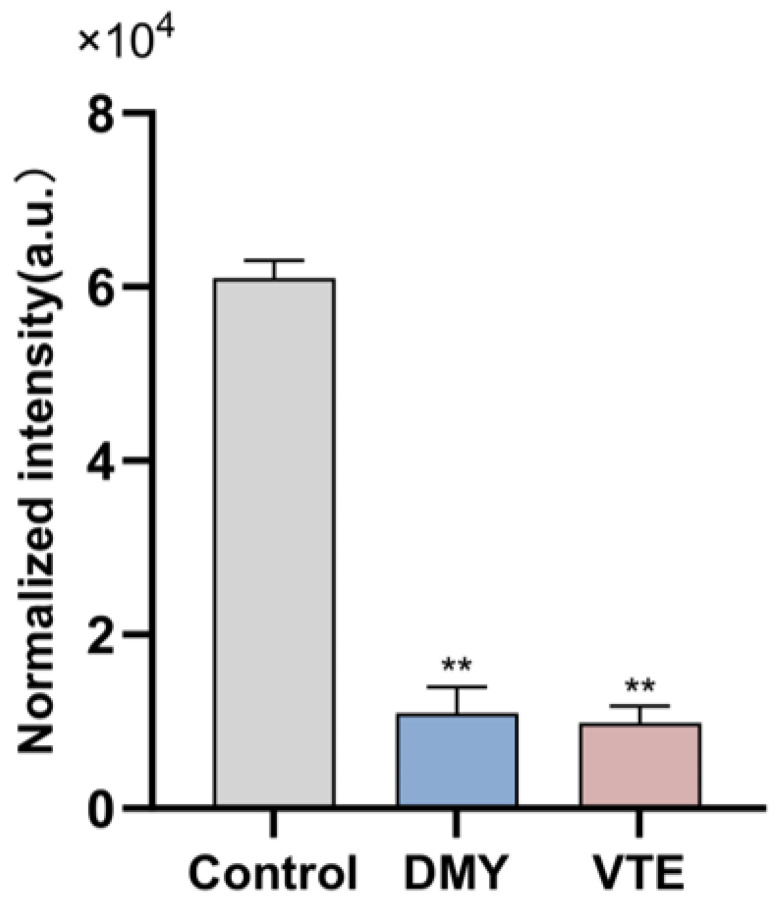
Relative abundance of Bacterial terpenols in *S. aureus* after DMY and VTE treatment. **: *p* < 0.01.

Isoprenoid-like lipids are mainly composed of terpenoids, which are built from isoprene units of varying carbon numbers. The C55-type isoprenoids composed of 11 isoprene units are bacterial terpenoids. As essential lipid carriers, they help transfer hydrophilic “Park” nucleotide intermediates across the hydrophobic membrane during cell wall synthesis and facilitate the incorporation of glycan monomers at sites of cell wall expansion. In this study, the relative abundance of bacterial terpenols in the control, DMY-treated, and VTE-treated groups was 6.1 × 10^4^, 1.1 × 10^4^, and 9.8 × 10^3^, respectively (Figure 10). Terpenol levels were significantly lower in both treatment groups than in the control group (*p* < 0.01). These results suggest that DMY and VTE may inhibit cell wall biosynthesis and suppress bacterial growth by restricting the transmembrane transport of Park nucleotide precursors and limiting monomer incorporation at cell wall expansion sites. This effect may be associated with an insufficient supply of lipid carriers or impaired carrier recycling.

In summary, DMY and VTE may exert antibacterial effects by regulating lipid metabolism and altering the cell wall and membrane of *S. aureus* through multiple pathways. DMY and VTE not only altered the cellular lipid composition and the relative abundance of lipid classes, but also increased membrane fluidity by increasing fatty acid unsaturation and shortening fatty acid chain length, which may disrupt membrane homeostasis. The decrease in terpenoid lipids further suggests that DMY and VTE may interfere with cell wall synthesis. In addition, DMY and VTE may promote the accumulation of glycerolipids which can inhibit bacterial growth. Together, these lipid changes provide further evidence that DMY and VTE mainly target the cell wall and membrane to inhibit *S. aureus*.

### 2.9. Evaluation of Selected Proteins by RT-qPCR

Based on the proteomics and lipidomics results, we selected three key cell wall/cell membrane–associated proteins (LytM, LtaS, and DgkB) and validated the transcription levels of their corresponding genes by RT–qPCR.

**Figure 11:**
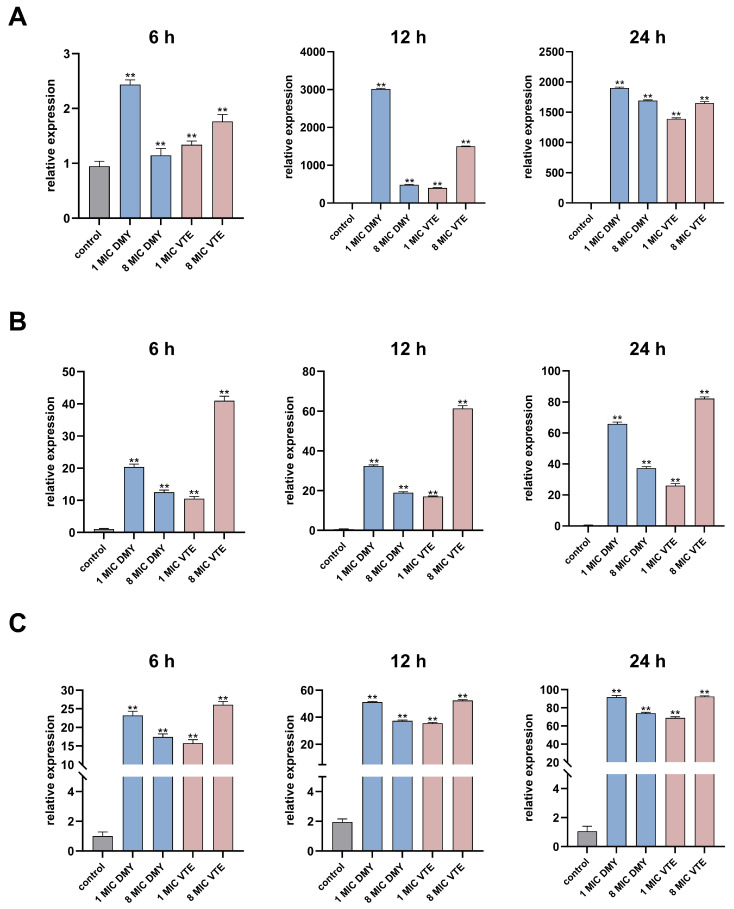
Effect of DMY and VTE on the expression of cell wall-related genes in *S. aureus*. (**A**) Expression levels of LytM after 6 h, 12 h, and 24 h of drug treatment. (**B**) Expression levels of LtaS after 6 h, 12 h, and 24 h of drug treatment. (**C**) Expression levels of DgkB after 6 h, 12 h, and 24 h of drug treatment. **: *p* < 0.01.

In *S*. *aureus*, lipoteichoic acid (LTA) synthesis is closely linked to the diacylglycerol kinase DgkB. During LtaS-mediated LTA synthesis, DAG is generated as a by-product [40,41]. Excessive DAG accumulation is detrimental to the bacterial cell. DgkB phosphorylates DAG to phosphatidic acid, thereby reducing DAG-associated toxicity and helping maintain membrane lipid homeostasis [85]. We measured the transcript levels of *lytM*, *ltaS*, and *dgkB* in *S. aureus* by RT-qPCR at 6, 12, and 24 h (Figure 11). All three genes were significantly upregulated, consistent with the proteomic results. For lytM, 1× MIC DMY and 8× MIC VTE produced the strongest induction. Upregulation of lytM after DMY and VTE treatment may enhance peptidoglycan hydrolysis and weaken the cell wall integrity of *S. aureus* [30]. In addition, 1× MIC DMY and 8× MIC VTE resulted in the highest *dgkB* transcript levels at all three time points, which was consistent with the trend observed for *ltaS*. These results suggest that DMY and VTE may respond to cell wall and membrane damage by increasing *ltaS* expression, which may raise DAG generation during lipoteichoic acid synthesis [36,37]. This increase may then trigger *dgkB* upregulation to convert DAG to phosphatidic acid and reduce DAG-associated stress [85].

Although we systematically investigated the antibacterial mechanisms of DMY and VTE against *S. aureus* using proteomic and lipidomic approaches, this study has limitations because the experimental design used a single concentration and single time point for each omics assay. Proteomic analysis was performed at 24 h, whereas lipidomic analysis was conducted at 6 h. These time points were selected to reflect the different response dynamics of proteins and lipids during antimicrobial exposure. Protein-level changes often require more time to accumulate, so the 24 h time point is more suitable for capturing longer-term bacterial responses. In contrast, lipidomic changes can occur rapidly, and membrane lipid alterations are often early indicators of stress. However, using only one concentration and one time point may not capture all stages of antimicrobial activity, because antimicrobial agents can induce different stress and adaptive responses over time. Therefore, our design limits the ability to distinguish primary antibacterial mechanisms from secondary stress responses. Future studies should include multiple time points and a range of concentrations in omics analyses to better characterize dynamic antimicrobial responses and to clarify the molecular mechanisms at different stages.

## 3. Materials and Methods

### 3.1. Materials

*S. aureus* strains were obtained from the China Medical Culture Collection Center (Beijing, China). Young vine tea leaves were harvested in Hunan Province, China. The leaves were air-dried, ground into powder, and sieved through a 30-mesh sieve. The vine tea powder was suspended in deionized water at a ratio of 1:10 (*w*/*v*) and soaked for 30 min at room temperature. The suspension was then extracted in a boiling water bath for 25 min. After extraction, the mixture was filtered to remove insoluble residues. The filtrate was concentrated under reduced pressure at 40 °C using a rotary evaporator and stored at 4 °C until use. Dihydromyricetin (purity ≥ 95%) was purchased from Beijing Banxia Biotechnology Co., Ltd. (Beijing, China).

### 3.2. Chemical Characterization of VTE

Flavonoids in vine tea extract were analyzed using ultra-high-performance liquid chromatography coupled with tandem mass spectrometry (UHPLC-MS/MS; Nexera UHPLC system and LCMS-8040, Shimadzu, Kyoto, Japan) as previously described [86]. Chromatographic separation was performed on an RP-C18 Inertsil ODS-4 analytical column (100 mm × 2.1 mm, 2 μm; GL Sciences Inc., Tokyo, Japan). The mobile phase consisted of water containing 10 mM ammonium formate and 0.1% (*v*/*v*) formic acid (A) and acetonitrile (B). The gradient program was as follows: 0–10 min, 5–20% B; 10–22 min, 20% B; 22–36 min, 20–50% B; 36–40 min, 95% B; and 40–50 min, 5% B. The column temperature was set at 35 °C. The flow rate was 0.25 mL/min, and the injection volume was 4 μL. Mass spectrometric detection was performed using an electrospray ionization (ESI) source operating in negative ion mode. Dihydromyricetin (purity ≥ 95%), dihydroquercetin (purity ≥ 90%), myricetin 3-O-rhamnoside (purity ≥ 98%) and myricetin (purity ≥ 96%) were used as reference standards for qualitative identification and quantitative analysis of the target flavonoids. All samples were analyzed in triplicate.

### 3.3. Mass Spectrometric Characterization of Proteins

DMY was dissolved in sterile deionized water, and VTE was prepared by extraction with deionized water. Both were diluted with nutrient broth to 1× MIC. *S. aureus* was treated with DMY or VTE at 1× MIC for 24 h. Untreated cells receiving an equal volume of sterile deionized water served as the control. Cells were collected by centrifugation at 14,000 rpm for 5 min at 4 °C. The pellets were washed three times with phosphate-buffered saline (PBS) and then freeze-dried. For protein extraction, 1 mL of lysis buffer (8 M urea, 2% (*m*/*v*) CHAPS, 62 mM dithiothreitol (DTT), and 1% phenylmethylsulfonyl fluoride (PMSF)) was added to 25 mg of freeze-dried biomass. The mixture was vortexed and incubated on ice, with vortexing for 10 s every 2 min to ensure complete lysis. The suspension was sonicated using a cell sonicator (Ningbo Saine Bio-Tech Co., Ltd., Ningbo, China) for 15 min with a pulse duration of 4 s. The lysate was then centrifuged, and the supernatant containing soluble proteins was collected. Protein concentration was determined using the Bradford assay. Equal amounts of protein were analyzed by SDS–PAGE to evaluate protein quality.

Proteins were precipitated by adding pre-chilled acetone at a volume ratio of 1:4 (sample:acetone) and incubating the mixture overnight at −20 °C. The precipitated proteins were collected by centrifugation at 14,000 rpm for 10 min and washed twice with pre-chilled acetone at −20 °C. After removing residual acetone, the protein pellets were resuspended in triethylammonium bicarbonate (TEAB) buffer. For digestion, dithiothreitol (DTT) was added to a final concentration of 5 mM, and samples were incubated at 56 °C for 30 min for reduction. Iodoacetamide (IAA) was then added to 10 mM, followed by incubation in the dark at room temperature for 30 min for alkylation. Lys-C was added at an enzyme-to-protein ratio of 1:50 (*w*/*w*), and digestion was performed at 37 °C for 3 h. Trypsin was then added at 1:50 (*w*/*w*), and digestion was continued at 37 °C for 12 h. Digestion was stopped by adding trifluoroacetic acid (TFA) to a final concentration of 0.5%. To correct for technical variation, 50 fmol of SILAC-labeled peptide (Thermo Fisher Scientific, Waltham, MA, USA) was added to each digested sample as an exogenous internal standard. Peptide mixtures were desalted using a Sep-Pak C18 column (Waters, Milford, MA, USA) and concentrated under vacuum (SpeedVac concentrator; Thermo Fisher Scientific, Waltham, MA, USA). Proteomic analysis was performed using a Waters ultra-high-performance liquid chromatography–mass spectrometry (UPLC–MS) platform. Peptides were separated on an ACQUITY UPLC M-Class system equipped with an HSS T3 C18 column (15 cm × 75 μm i.d.). Mobile phase A was 0.1% formic acid (FA) in water, and mobile phase B was 0.1% FA in acetonitrile. The flow rate was 0.3 μL/min. Mass spectrometric analysis was performed on a SYNAPT G2-Si high-definition mass spectrometer (Waters Corporation, Milford, MA, USA). Data were acquired in high-definition mass spectrometry (HDMS) mode using an electrospray ionization (ESI) source (Thermo Fisher Scientific, Waltham, MA, USA) operated in positive ion mode.

Raw data were processed using MassLynx 4.2 SCN 1050, and quantitative performance was assessed using MassPREP™ standards. Protein identification and differential expression analysis were performed using Progenesis QI for Proteomics. Database searches were conducted against UniProt, with results filtered at a 1% false discovery rate (FDR). All experiments were performed with three independent biological replicates.

### 3.4. Lipidomic Analysis

Cell treatment and harvesting were performed as described in the protein sample preparation section, except that the exposure time was 6 h. Cells were collected by centrifugation at 4 °C and washed twice with PBS (0.1 M, pH 7.2). The pellets were then lyophilized. For lipid extraction, the dried pellets were resuspended in chloroform/methanol (2:1, *v*/*v*). Immediately after resuspension, 10 μL of SPLASH^®^ LIPIDOMIX quantitative internal standard mixture (Avanti Polar Lipids, Alabaster, AL, USA) was added to each sample. Samples were homogenized using an ultrasonic cell disruptor (Ningbo Sainezi Biotechnology Co., Ltd., Ningbo, China) with intermittent pulses (2 s) at 3% power. The homogenates were centrifuged, and the supernatants were collected and concentrated to a final volume of 100 μL. Liquid chromatography was performed on a Waters UPLC system equipped with a CSH C18 column (1.7 μm, 2.1 mm × 100 mm). Mobile phase A was acetonitrile/water (4:6, *v*/*v*) containing 0.1% formic acid and 10 mM ammonium formate, and mobile phase B was acetonitrile/isopropanol (9:1, *v*/*v*) containing 0.1% formic acid and 10 mM ammonium formate. The flow rate was 0.3 mL/min, the injection volume was 2 μL, and the column temperature was 50 °C. Mass spectrometric analysis was performed using a Waters (Milford, MA, USA) Xevo TQ-S triple quadrupole mass spectrometer. Data were acquired over an *m*/*z* range of 50–1200. Nitrogen was used as the gas for all gas pathways. The capillary voltage was 3200 V in positive ion mode and 2500 V in negative ion mode. The cone voltage was 35 V. The desolvation temperature was 400 °C with a gas flow rate of 800 L/h, and the cone gas flow rate was 50 L/h. The ion source temperature was 120 °C.

Data were acquired using MassLynx 4.1 (Waters Corporation, Milford, MA, USA). Raw data were processed in Progenesis QI 2.0 and EZinfo 3.0 (Waters Corporation) for peak picking, alignment, and normalization using SPLASH^®^ LIPIDOMIX internal standards. The resulting data matrix was uploaded to MetaboAnalyst (v5.0) for further statistical analysis and visualization. Lipid species were annotated by matching accurate masses and MS/MS fragmentation patterns against the LipidMaps database. Normalized intensities (a.u.) were calculated by correcting peak intensities with SPLASH^®^ LIPIDOMIX internal standards and normalizing to dry cell weight (DW). All analyses were performed with three independent biological replicates, and the FDR was controlled at 1%.

### 3.5. Bioinformatics Analysis

Differentially expressed proteins (DEPs) were functionally annotated using the UniProt database. Gene Ontology (GO) analysis was used to classify DEPs into three categories: biological process, cellular component, and molecular function. Pathway enrichment analysis was performed using the Kyoto Encyclopedia of Genes and Genomes (KEGG) database.

### 3.6. Total RNA Extraction and Quantitative Real-Time Fluorescent PCR for Bacteria

Total RNA was extracted from *S. aureus* cells using TRIzol reagent. Bacterial pellets stored at −80 °C were mixed with TRIzol and vortexed thoroughly. The mixture was incubated at room temperature for 10 min and centrifuged at 12,000 rpm for 5 min at 4 °C. The supernatant was transferred to a new tube, and 0.3 mL chloroform was added. After gentle mixing, the sample was incubated at room temperature for 15 min and centrifuged at 12,000 rpm for 15 min at 4 °C. After phase separation, 0.4 mL of the aqueous phase was transferred to a fresh tube, and 0.5 mL isopropanol was added to precipitate RNA. The mixture was incubated at room temperature for 10 min and centrifuged at 12,000 rpm for 10 min at 4 °C. The RNA pellet was washed with 1 mL of 75% (*v*/*v*) RNase-free ethanol and incubated on ice for 10 min, followed by centrifugation at 8000 rpm for 5 min at 4 °C. The supernatant was discarded, and the pellet was air-dried at room temperature for 10 min. The RNA was dissolved in 50 μL RNase-free water and incubated at 4 °C for 3 h to ensure complete dissolution. First-strand cDNA was synthesized using a FastQuant RT Kit (with gDNase; TIANGEN BIOTECH (BEIJING) Co., Ltd., Beijing, China) according to the manufacturer’s instructions. Quantitative real-time PCR was performed using the SuperReal PreMix Plus (SYBR Green) kit (TIANGEN BIOTECH (BEIJING) Co., Ltd., Beijing, China). Reaction mixtures were prepared on ice. Primer sequences and cycling conditions are provided in Table 2. Remaining cDNA was stored at −20 °C until use.

## 4. Conclusions

**Figure 12:**
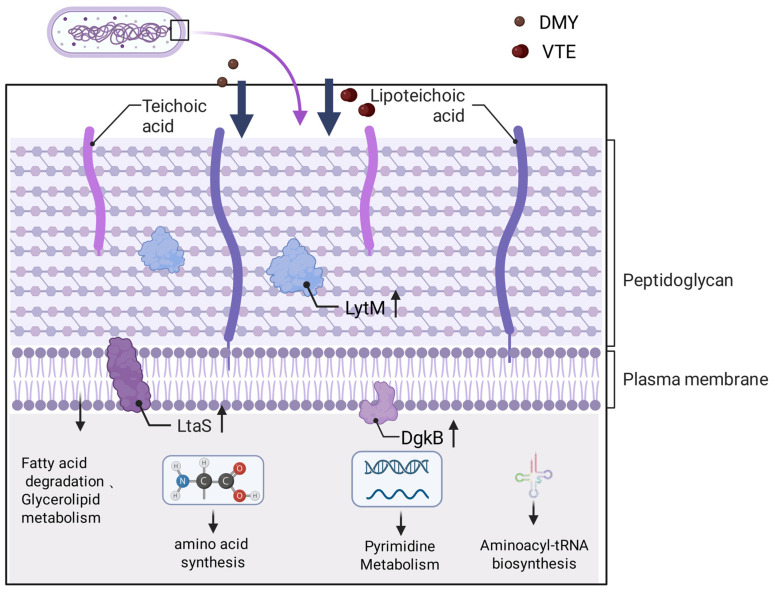
Antibacterial mechanism of DMY and VTE against *S. aureus*. Arrows point to the corresponding pathway names/labels. Upward arrows denote increased expression/abundance.

This study explored the antibacterial mechanisms of DMY and VTE against *S*. *aureus* using proteomics, lipidomics, and RT–qPCR. The results suggest that the antibacterial mechanisms of DMY and VTE are mainly linked to disruption of cell wall–membrane homeostasis. After DMY or VTE exposure, *S. aureus* showed increased phospholipid fatty acid unsaturation, shorter acyl chain length, and higher DAG levels. These changes indicate increased membrane fluidity and structural perturbation. Accordingly, cell wall-related proteins (e.g., LytM, SceD, and LtaS) were upregulated, suggesting activation of compensatory stress and repair responses following cell wall damage. In addition, the increased abundance of lipase and enzymes involved in fatty acid degradation suggests that damaged membrane lipids may be further hydrolyzed and converted to acetyl-CoA, which could support stress adaptation and membrane remodeling. VTE also altered proteins involved in pyrimidine metabolism and protein synthesis, which may further strengthen its antibacterial activity. (Figure 12) Overall, DMY and VTE appear to inhibit *S. aureus* growth primarily by disturbing cell wall and membrane homeostasis.

## Figures and Tables

**Table 1:** Identification and quantification of major flavonoid compounds in VTE.

Peak No.	Compound	RT (min)	Quantifier Transition (*m*/*z*)	Qualifier Transition (*m*/*z*)	Ion Ratio	Concentration (μg/mL)
1	Dihydromyricetin	3.29	319.3/193.0	319.3/125.1	0.2724 (0.2498)	2780.237
2	Dihydroquercetin	3.38	303.1/151.0	303.1/175.0	0.6823 (0.6235)	157.658
3	Myricetin-3-O-rhamnoside	3.40	463.1/316.0	463.1/271.0	0.3520 (0.3605)	308.769
4	Myricetin	3.46	317.0/151.0	317.0/179.0	0.7162 (0.7429)	0.589

RT, retention time (min); transitions are shown as precursor/product ion pairs (*m*/*z*); the quantifier transition was used for quantification, and the qualifier transition was used for identity confirmation; ion ratio, qualifier/quantifier (measured; reference value from standards shown in parentheses); concentration, analyte concentration in the sample solution (μg/mL).

**Table 2:** Primer sequences used for RT-qPCR.

Target Gene	Primer Name	Primer Sequence 5′–3′
*l* *ytM*	LytM-F	ACGGTGTCGACTATGCAATGC
	LytM-R	TACTTGATTGCCGCCACCA
*l* *taS*	LtaS-F	TTAGCCAACTGAATCTGC
	LtaS-R	GATGCCTCTTTCACTTTT
*d* *gkB*	DgkB-F	CCGCTCCAATGCTCCCCCTT
	DgkB-R	CACGTCGTACGTCAGCTCCG

F, forward primer; R, reverse primer. All primer sequences are presented in the 5′-3′direction.

## Data Availability

The original contributions presented in this study are included in the article. Further inquiries can be directed to the corresponding author.
