# Correction: Hui et al. Omics Reveals the Antibacterial Mechanism of Dihydromyricetin and Vine Tea Extract Against *Staphylococcus aureus* via Cell Wall and Membrane Disruption. *Molecules* 2026, *31*, 313

**DOI:** 10.3390/molecules31071081

**Published:** 2026-03-26

**Authors:** Qiaoni Hui, Ting Li, Keke He, Wei Ma, Ying Guo, Yao Zhang, Liya Song

**Affiliations:** Department of Cosmetics, School of Light Industry Science and Engineering, Beijing Technology and Business University, Beijing 100048, China; huiqiaoni1111@163.com (Q.H.); qianandeliting@126.com (T.L.); 13121516520@163.com (K.H.); mawei901@163.com (W.M.); gy11072000@163.com (Y.G.); jiajingwenzhangyao@126.com (Y.Z.)

## 1. Error in Figure/Table

In the original publication [1], there was a mistake in Figure 1 and Table 1. The chemical characterization of vine tea extract (VTE) was incorrectly presented in the published version. Figure 1 and Table 1, and the associated text have been corrected to reflect the liquid chromatography-tandem mass spectrometry (LC-MS/MS) analysis used in this study. The corrected Figure 1 and Table 1 appear below. 

## 2. Missing Citation

In the original publication, the reference “Yilmaz, M.A.; Ertas, A.; Yener, I.; Akdeniz, M.; Cakir, O.; Altun, M.; Demirtas, I.; Boga, M.; Temel, H. A Comprehensive LC-MS/MS Method Validation for the Quantitative Investigation of 37 Fingerprint Phytochemicals in Achillea Species: A Detailed Examination of *A. coarctata* and *A. monocephala*. *J. Pharm. Biomed. Anal.* **2018**, *154*, 413–424” was not cited. The citation has now been inserted into the LC-MS/MS method description in Section 3.2 as Reference 86 [2].

## 3. Text Correction

There were errors in the original publication in Section 2.1, Characterization of Major Flavonoids in Vine Tea Extract, and Section 3.2, Chemical Characterization of VTE.

A correction has been made to Section 2.1, Characterization of Major Flavonoids in Vine Tea Extract:

We characterized the major flavonoids in VTE using liquid chromatography-tandem mass spectrometry (LC-MS/MS). The overlaid multiple reaction monitoring (MRM) chromatograms are shown in Figure 1. Based on the LC-MS/MS data (Figure 1 and Table 1), VTE contained a relatively high level of flavonoids. Four major flavonoids were detected and identified: dihydromyricetin, dihydroquercetin, myricetin 3-O-rhamnoside, and myricetin. Among them, DMY was the predominant bioactive component.

A correction has also been made to Section 3.2, Chemical Characterization of VTE:

Flavonoids in vine tea extract were analyzed using ultra-high-performance liquid chromatography coupled with tandem mass spectrometry (UHPLC-MS/MS; Nexera UHPLC system and LCMS-8040, Shimadzu, Kyoto, Japan) as previously described [86]. Chromatographic separation was performed on an RP-C18 Inertsil ODS-4 analytical column (100 mm × 2.1 mm, 2 μm; GL Sciences Inc., Tokyo, Japan). The mobile phase consisted of water containing 10 mM ammonium formate and 0.1% (*v*/*v*) formic acid (A) and acetonitrile (B). The gradient program was as follows: 0–10 min, 5–20% B; 10–22 min, 20% B; 22–36 min, 20–50% B; 36–40 min, 95% B; and 40–50 min, 5% B. The column temperature was set at 35 °C. The flow rate was 0.25 mL/min, and the injection volume was 4 μL. Mass spectrometric detection was performed using an electrospray ionization (ESI) source operating in negative ion mode. Dihydromyricetin (purity ≥ 95%), dihydroquercetin (purity ≥ 90%), myricetin 3-O-rhamnoside (purity ≥ 98%) and myricetin (purity ≥ 96%) were used as reference standards for qualitative identification and quantitative analysis of the target flavonoids. All samples were analyzed in triplicate.

## 4. References

Reference 86 [2] has been added. With this correction, the order of all references has been adjusted accordingly.

The authors state that the scientific conclusions are unaffected. This correction was approved by the Academic Editor. The original publication has also been updated.

## Figures and Tables

**Figure 1:**
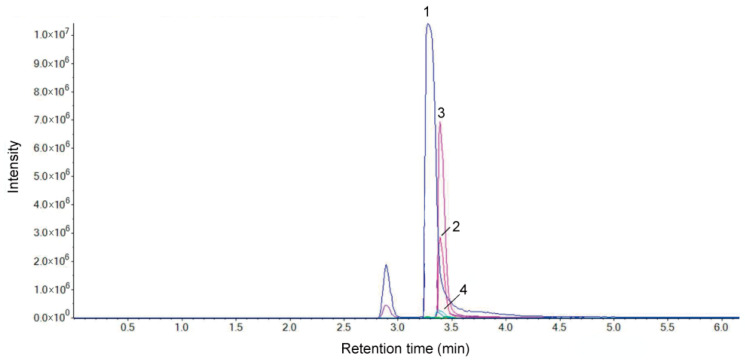
Overlaid MRM chromatograms of VTE: Peak 1: dihydromyricetin; Peak 2: dihydroquercetin; Peak 3: myricetin-3-O-rhamnoside; Peak 4: myricetin.

**Table 1:** Identification and quantification of major flavonoid compounds in VTE.

Peak No.	Compound	RT (min)	Quantifier Transition (*m*/*z*)	Qualifier Transition (*m*/*z*)	Ion Ratio	Concentration (μg/mL)
1	Dihydromyricetin	3.29	319.3/193.0	319.3/125.1	0.2724 (0.2498)	2780.237
2	Dihydroquercetin	3.38	303.1/151.0	303.1/175.0	0.6823 (0.6235)	157.658
3	Myricetin-3-O-rhamnoside	3.40	463.1/316.0	463.1/271.0	0.3520 (0.3605)	308.769
4	Myricetin	3.46	317.0/151.0	317.0/179.0	0.7162 (0.7429)	0.589

RT, retention time (min); transitions are shown as precursor/product ion pairs (*m*/*z*); the quantifier transition was used for quantification, and the qualifier transition was used for identity confirmation; ion ratio, qualifier/quantifier (measured; reference value from standards shown in parentheses); concentration, analyte concentration in the sample solution (μg/mL).
